# Supplementary material for: Collider scope: when selection bias can substantially influence observed associations
Source: Int J Epidemiol. 2017 Sep 27;47(1):226–35. doi: 10.1093/ije/dyx206 (PMC5837306; doi:10.1093/ije/dyx206)
Supplement: Supplementary Data [file dyx206_ije-2016-10-1194-file002.docx]

**Supplementary Methods**

Genotyping in the ALSPAC mothers was carried out on the Illumina human660W-quad array. Quality control was carried out using PLINK (v1.07) (1). This removed SNPs and individuals with low genotyping rates (>5%), SNPs with a Hardy-Weinberg equilibrium P value of less than 1.0e-06 and samples showing evidence of population stratification. SNPs were imputed using Impute2 v2.2.2 to the 1000 genomes phase 1 version 3 imputation panel.

We constructed genetic risk scores for smoking initiation (i.e., ever vs never smoking) in PLINK using SNPs associated with smoking initiation at a P-value threshold of < 0.05 in the Tobacco and Genetics consortium GWAS (N=154,174) (2). LD clumping was performed using an r-squared threshold of 0.1, resulting in 18,011 variants being used in the analysis. Risk scores were weighted by multiplying each allele by the magnitude of association with ever smoking (log odds ratio) from the TAG GWAS and were converted to z-scores.

Smoking initiation (ever vs never smoking) and maternal education were ascertained from questionnaires administered at 18 weeks and 32 weeks gestation respectively. Maternal education was recoded to a binary variable indicating whether or not the mother had a degree. Analyses were conducted in Stata (version 14.1). Associations between genetic risk scores, smoking initiation and maternal education were assessed using logistic regression.

1. Purcell S., Neale B., Todd-Brown K., Thomas L., Ferreira M. A., Bender D. et al. PLINK: a tool set for whole-genome association and population-based linkage analyses, American Journal of Human Genetics 2007: 81: 559-575.

2. Furberg H., Kim Y., Dackor J., Boerwinkle E., Franceschini N., Ardissino D. et al. Genome-wide meta-analyses identify multiple loci associated with smoking behavior, Nature Genetics 2010: 42: 441-U134.
